# Effect of Microwave-Assisted Synthesis and Sintering of Lead-Free KNL-NTS Ceramics

**DOI:** 10.3390/ma15113773

**Published:** 2022-05-25

**Authors:** Anggel Lagunas-Chavarría, María Guadalupe Navarro-Rojero, María Dolores Salvador, Rut Benavente, Jose Manuel Catalá-Civera, Amparo Borrell

**Affiliations:** 1 Instituto de Tecnología de Materiales (ITM), Universitat Politècnica de València, Camino de Vera, s/n, 46022 Valencia, Spain; danny.lagunas09@anahuac.mx (A.L.-C.); dsalva@mcm.upv.es (M.D.S.); aborrell@upv.es (A.B.); 2 Advance Technology Centre (CIATEQ A.C.), Av. del Retablo 150, Col. Constituyentes Fovissste, Querétaro 76150, Mexico; maria.navarro@ciateq.mx; 3 Instituto de las Tecnologías de la Información y Comunicaciones (ITACA), Universitat Politècnica de València, Camino de Vera, s/n, 46022 Valencia, Spain; jmcatala@dcom.upv.es

**Keywords:** KNL-NTS ceramics, synthesis, calcination, microwave processing, piezoelectric properties

## Abstract

Lead-free piezoelectric powders (K_0.44_Na_0.52_Li_0.04_)(Nb_0.82_Ta_0.10_Sb_0.04_)O_3_ were obtained by conventional and microwave-assisted reactive heating. Firstly, the synthesis of the material was carried out following the mixed oxide route and employing both traditional methods and microwave technology. Thermogravimetry, X-ray diffraction, field emission scanning electron microscopy and electrical properties analyses were evaluated. X-ray diffraction of the powders calcined by the microwave process shows the formation of perovskite structure with orthorhombic geometry, but it is possible to observe the presence of other phases. The presence of the secondary phases found can have a great influence on the heating rate during the synthesis on which the kinetics of the reaction of formation of the piezoelectric compound depend. The calcined powder was sintered at different temperatures by conventional and non-conventional processes. The microstructure of the ceramics sintered by microwave at 1050 °C for 10 min shows perovskite cubes with regular geometry, of size close to 2–5 µm. However, the observed porosity (~8%), the presence of liquid phase and secondary phases in the microstructure of the microwave sintered materials lead to a decrease of the piezoelectric constant. The highest d_33_ value of 146 pC/N was obtained for samples obtained by conventional at 1100 °C 2 h compared to samples sintered by microwave at 1050 °C 10 min (~15 pC/N).

## 1. Introduction

Industry demands new methods of processing materials in order to improve their properties. Priority requirements in new processes involve the reduction of energy consumption and processing time. In that regard, microwave radiation heating has shown some significant advantages over conventional heating [1,2,3]. Microwave heating is a non-conventional technique that involves high heating rates and rapid processing times [4,5,6]. The properties of most materials heated by microwave radiation showed similar or superior properties to those obtained by conventional processing [7,8,9].

Materials such as inorganic compounds have been synthetized successfully by applying different microwave heating techniques [10,11,12,13,14]. However, the sintering of piezoelectric materials by microwave of energy remains without substantial research [15,16,17]. So far, there is a lack of literature about the use of microwave radiation to process piezoelectric materials, and exploration is, therefore, needed.

The good piezoelectric properties of K_0.5_Na_0.5_NbO_3_ (KNN) make it one of the most promising ceramics to replace lead-based piezoelectrics [18,19,20]. Recent studies have shown that the addition of Li and Ta improve these properties [21,22]. In this work, the material (K_0.44_Na_0.52_Li_0.04_)(Nb_0.82_Ta_0.10_Sb_0.04_)O_3_ (KNL-NTS) was selected to test the benefits that microwave technology offers.

It is well known that obtaining piezoelectric ceramic powder by the traditional solid-state reaction method from precursors is the most widespread. Precursor oxides calcination at temperatures 700–800 °C for several hours is an effective method; however, it entails long processing times as well as high energy costs. An alternative is the use of microwave radiation as a softer technique for obtaining these materials. The solid-state reaction assisted by microwave heating has led to the production of KNN systems at lower temperatures than conventional methods without the presence of secondary phases [23]. It can be expected that improving microwave heating by using single-mode systems instead of multi-mode systems will open up the options for this technology [24].

Another fundamental factor determining the properties of KNL-NTS is the sintering process. The ferroelectric, dielectric and piezoelectric properties are determined by the structural heterogeneities caused during sintering. Density, grain size, and formation of second phases depend on the sintering temperature and time, as well as on the chosen sintering method. During microwave heating, the material transforms the absorbed radiation into volumetric heat, sintering from within the individual grains. In this way, densification is favored and the process can be completed before the grains grow [9].

In this study, the feasibility of using microwave radiation in both reactive synthesis and sintering of KNL-NTS is analyzed. For this purpose, a single-mode microwave equipped with a 1 kW magnetron operating at a resonant frequency of 2.45 GHz in the TE_111_ heating mode has been employed. The materials obtained after the calcination and sintering process were analyzed by X-ray diffraction and high-resolution electron microscopy. The ferroelectric, dielectric and piezoelectric properties of these materials were determined and compared with their counterparts obtained by conventional techniques.

## 2. Materials and Methods

### 2.1. Starting Materials

The precursors used were Nb_2_O_5_ (99.9% purity, Sigma-Aldrich, St. Louis, MO, USA), Ta_2_O_5_ (99.0% purity, Sigma-Aldrich), Sb_2_O_5_ (99.9% purity, Alfa Aesar, Haverhill, MA, USA), Na_2_CO_3_ (99.5% purity, Panreac M&E, Barcelona, Spain), K_2_CO_3_ (99.0% purity, Fisher Scientific, Hampton, NH, USA) and Li_2_CO_3_ (99.5% purity, Panreac M&E). These precursors were attrition-milled individually in a Fritsch Pulverisette 7 planetary ball using ZrO_2_ stabilized with Y_2_O_3_ balls in ethanol during 2 h. This grinding step homogenized the particle size of the different powders between 2 and 10 μm. The powders obtained were oven dried at 80 °C for 24 h, were mixed according to the stoichiometry of the (K_0.44_Na_0.52_Li_0.04_)(Nb_0.82_Ta_0.10_Sb_0.04_)O_3_ formula and were attrition-milled again for 2 h.

### 2.2. Powder Calcination

The powder mixture obtained was thermogravimetrically analysed (TGA) up to 900 °C in air using different heating rates; 3, 5, 20, 30 and 40 °C·min^−1^, using a Q500 thermogravimetric analyzer (TA Instruments, New Castle, DE, USA).

**Figure 1:**
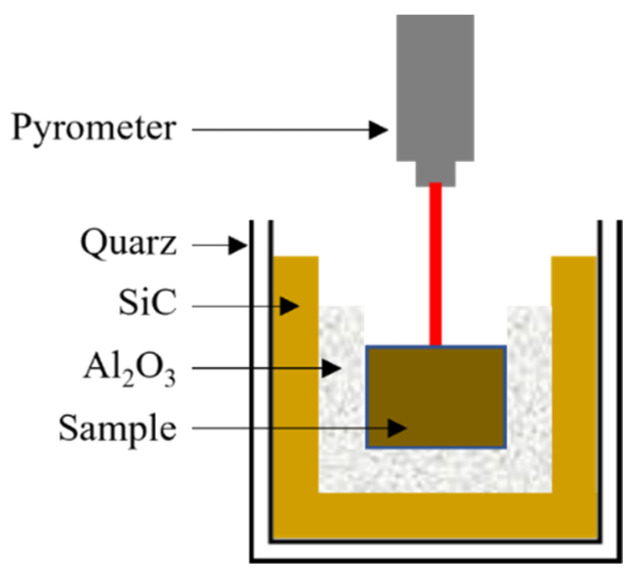
Schematic of the microwave cavity for calcination and sintering of KNL-NTS powders.

The calcination of mixed powders was carried out by conventional methods in air at 700, 750 and 800 °C with a heating rate of 3 °C·min^−1^ and a dwell time of 2 h, and microwave radiation in air at 650, 700, 750 and 800 °C with a heating rate of 30 °C·min^−1^ and a dwell time of 10 min. The microwave calcination temperature was measured directly on the powders using an optical pyrometer (CT-Laser 2MH, Optris GmbH, Berlin, Germany) and the previously calculated emissivity value of 0.833. Figure 1 shows the scheme of the microwave cavity, where the different layers used are observed: alumina fiber as a thermal insulator to protect the equipment, silicon carbide as an absorber material of excess microwave radiation and quartz as a sample holder.

### 2.3. Sintering Processes

(K_0.44_Na_0.52_Li_0.04_)(Nb_0.82_Ta_0.10_Sb_0.04_)O_3_ powders conventionally-synthesized at 800 °C for 2 h were uniaxially pressed al 80 MPa. Cylindrical specimens of 10 mm diameter and about 5 mm height were prepared and sintered by conventional and microwave processes. The conventional sintering (Carbolite Gero, HTF 1800) was carried out at 1100 °C with 2 h and 16 h of dwell time and 5 °C·min^−1^ of heating rate. Microwave sintering used a single mode cylindrical cavity operating in the TE_111_ mode with a resonant frequency of 2.45 GHz [9]. The samples were sintered at 950, 1000 and 1050 °C using a heating rate of 30 °C·min^−1^ with 10 min of holding time at the maximum temperature. The emissivity of the material at different temperatures, needed to control the temperature, was determined prior to sintering (0.837 at 950 °C, 0.841 at 1000 °C and 0.843 at 1050 °C).

### 2.4. Characterization Methods

In order to characterize and compare the powders calcined by the two methods used, X-ray diffraction and microstructural characterization were carried out.

The conventionally and non-conventionally sintered materials were characterized structurally, microstructurally, ferroelectrically, piezoelectrically and dielectrically. The techniques, equipment and conditions used are specified below.

The X-ray diffraction measurements (Bruker D8 Advance A25 diffractometer, Cu Ka radiation, Billerica, MA, USA) were performed in the 20–70° range with a step size of 0.02° and a reading time of 0.3 s.

The bulk density was measured using Archimedes’ principle by immersing the sample into a water-based liquid (ASTM C373-88). 

A field emission-scanning electron microscopy equipped with energy-dispersive analysis (FE-SEM, GEMINI ULTRA 55 MODEL, ZEISS, Jena, Germany) was used to determine the morphology of the calcined powders and sintered samples. Grain size was measured from the micrographs with an image analysis program. The grain size values presented corresponded to the average of 50 grains measured in each sample.

The ferroelectric properties of the sintered materials were measured with Radiant Technologies equipment (Inc. RT600HVS, Navi Mumbai, India). Samples were previously polished to obtain parallel surfaces and then an electrode with silver paste was applied at high temperature. 

Hysteresis loops were performed in a silicon oil bath at room temperature with a measured voltage of 3000 V. The parameter d33 was measured by placing the samples in a Berlincourt quasi-static meter. A low frequency pressure (100 Hz) was applied. 

The dielectric constant was measured at room temperature using a 4294A Precision Impedance Analyzer (Agilent, Santa Clara, CA, USA) from 0 to 100 MHz.

## 3. Results

**Figure 2:**
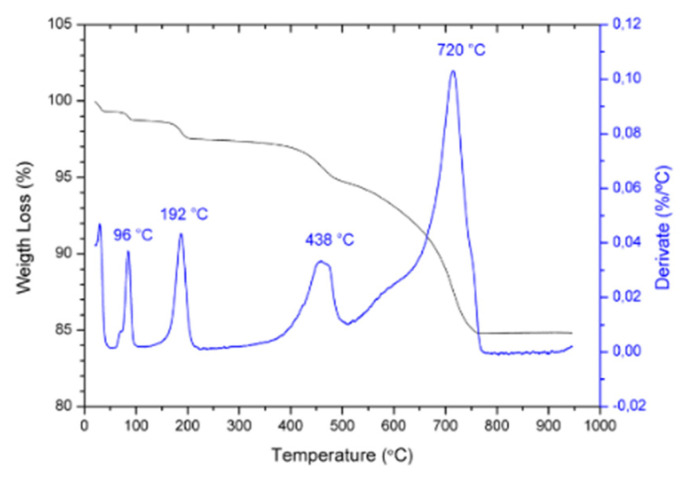
Thermogravimetric analysis (weigth loss in black and its derivate in blue) of the KNL−NTS powder mixture before calcination.

Thermogravimetric analysis was performed on the (K_0.44_Na_0.52_Li_0.04_)(Nb_0.82_Ta_0.10_Sb_0.04_)O_3_ (KNL-NTS) powder mixture prior to calcination. Figure 2 shows the thermogravimetric spectra and the corresponding derivative from room temperature up to 900 °C; the heating rate used was 3 °C·min^−1^. Four weight losses are observed at the temperatures of 96, 192, 438 and 720 °C.

**Figure 3:**
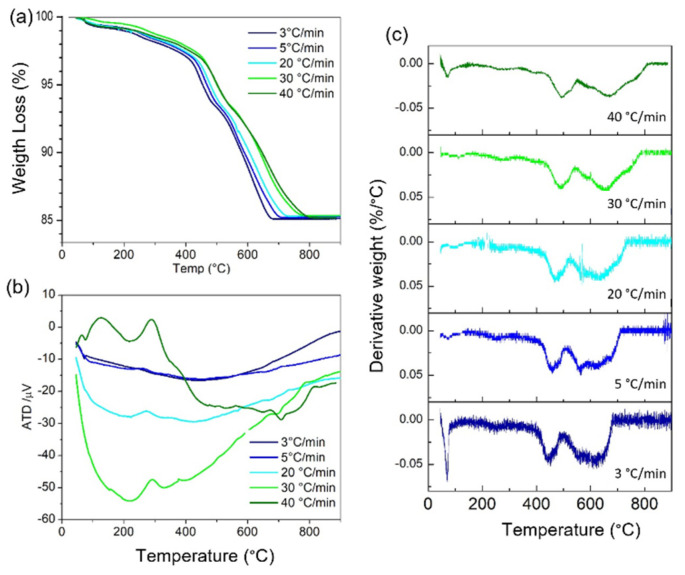
(**a**) Thermogravimetric analysis (TGA), (**b**) derivative and (**c**) differential thermal analysis of the KNL−NTS precursor mixture at different heating rates.

In order to determine the calcination reaction by the microwave process, a study of the influence of the heating rate on the thermogravimetric analysis was previously carried out. Figure 3 shows the TGA curves and derivatives performed on the uncalcined KNL−NTS powder mixture at 3, 5, 20, 30 and 40 °C·min^−1^ of heating rates from room temperature up to 900 °C.

**Figure 4:**
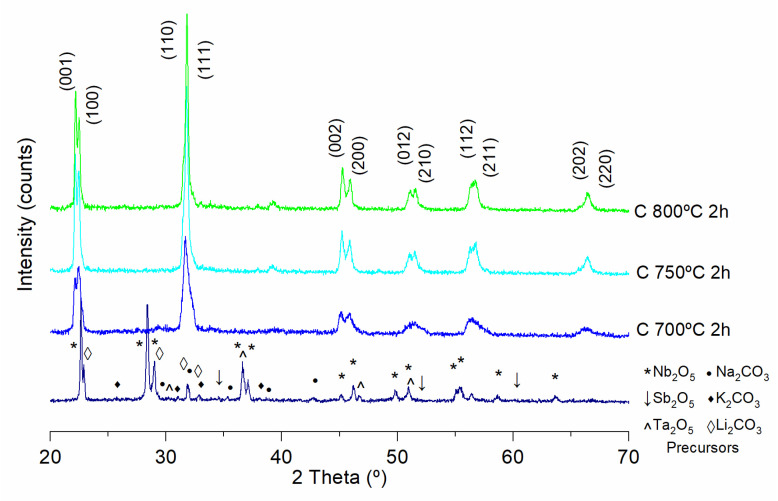
X-ray diffraction of KNL-NTS powders synthesized by conventional process at different temperatures.

In Figure 4, the X-ray diffraction patterns of powders calcined by the conventional process at 700, 750 and 800 °C during 2 h dwell time using a heating rate of 3 °C·min^−1^ are shown. In the lower part of the graph, the peaks corresponding to precursors, oxides and carbonates are observed.

**Figure 5:**
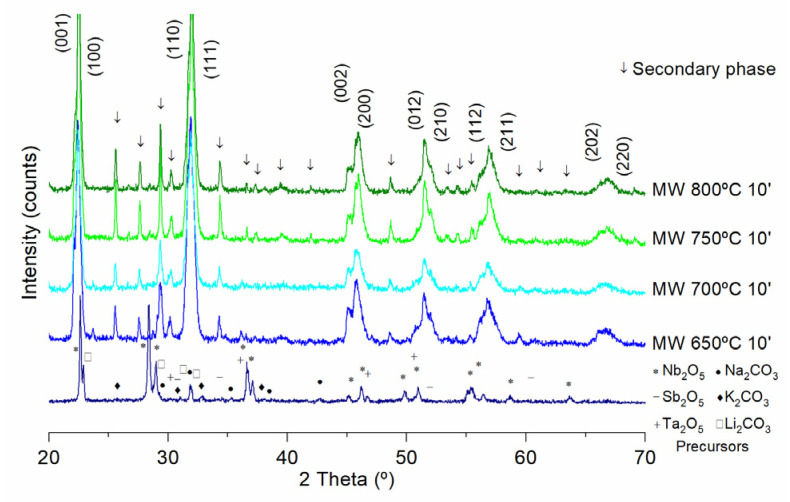
X-ray diffraction of KNL-NTS powders synthesized by microwave technology at different temperatures.

Figure 5 shows the X-ray diffraction peaks of the KNL-NTS powder calcined by microwave technology at 650, 700, 750 and 800 °C with a dwell time of 10 min at the maximum temperature and a heating rate of 30 °C·min^−1^. The peaks corresponding to precursors, oxides and carbonates are observed in the lower part of the graph.

**Figure 6:**
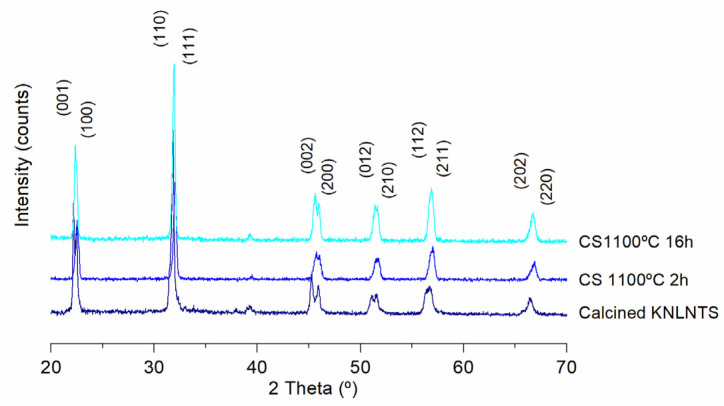
X-ray diffraction of KNL-NTS samples sintered by conventional process at different dwell times.

Sintering of the KNL-NTS materials was carried out from powders calcined at 800 °C for 2 h by the conventional method [25,26]. This choice is due to the previous study where it has been shown that under these conditions the pure perovskite phase predominates and there is no formation of secondary phases. Conventional sintering of KNL-NTS powder was carried out at 1100 °C for 2 h and 16 h of dwell time and 5 °C·min^−1^ of heating rate; these parameters are within the range established in the literature [27,28,29]. Figure 6 shows the X-ray diffraction patterns of KNL-NTS powders sintered by conventional process.

**Figure 7:**
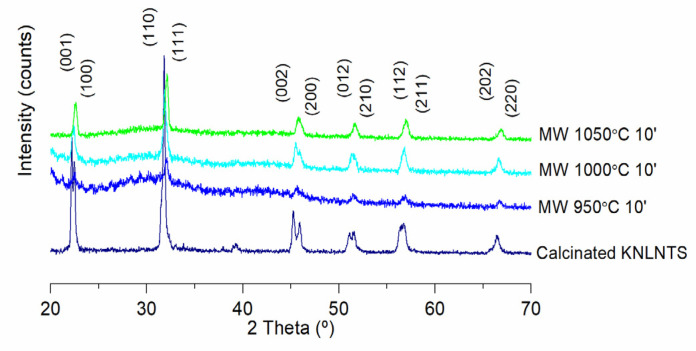
X-ray diffraction of the KNL-NTS samples sintered by microwave at different temperatures.

Figure 7 shows the X-ray diffraction patterns of KNL-NTS powders (previously calcined at 800 °C 2 h by conventional route) sintered by microwave at different temperatures, namely, 950, 1000 and 1050 °C with 10 min dwell time and a heating rate of 30 °C·min^−1^. The maximum sintering temperature when applying microwave technology was 1050 °C, since when this temperature is exceeded, the sample starts to melt.

Table 1 shows the different values obtained for the physical and electrical properties of the KNL-NTS materials obtained by both sintering methods as a function of the sintering conditions. The theoretical density of these materials has been determined by helium pycnometry, giving a value of 4.72 g·cm^−1^.

**Figure 8:**
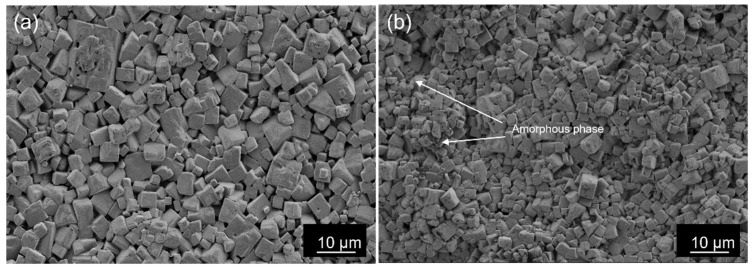
FE-SEM images of KNL-NTS samples sintered by: (**a**) conventional at 1100 °C 16 h and (**b**) microwave at 1050 °C 10 min.

The conventionally and microwave sintered KNL-NTS samples were analyzed by FE-SEM. The images obtained (Figure 8) show the typical cuboidal structures of perovskite.

**Figure 9:**
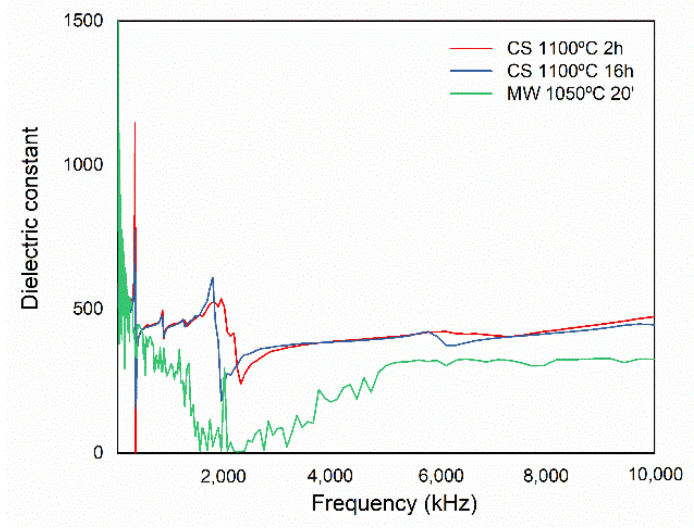
Dielectric constant values of KNL-NTS samples sintered by conventional and microwave.

The room temperature dielectric constant of ceramics sintered by the conventional method at 1100 °C for 2 and 16 h and by microwave at 1050 °C for 20 min was measured. The results are shown in Figure 9.

**Figure 10:**
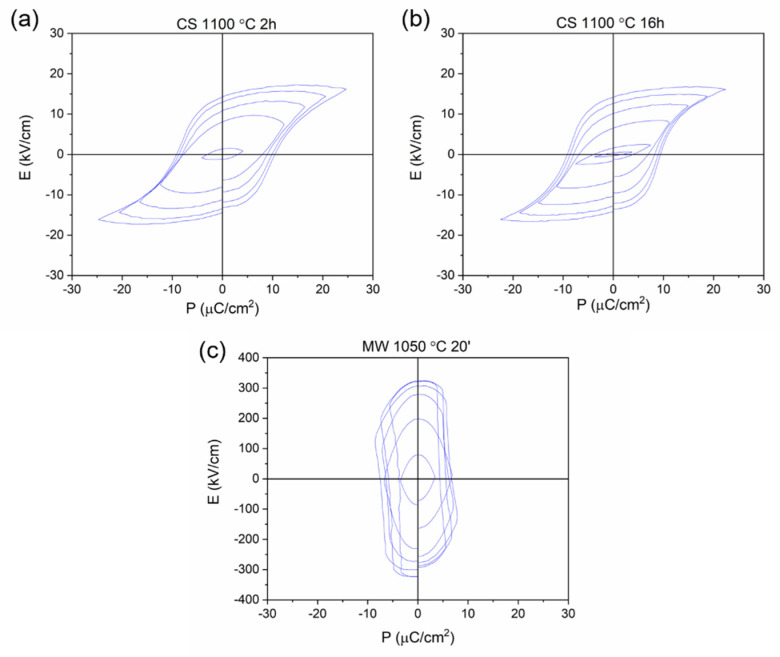
Electrical field hysteresis loops of KNL−NTS samples sintered by: (**a**) conventional methods at 1100 °C 2 h, (**b**) 1100 °C 16 h and (**c**) microwave 1050 °C 20 min.

The hysteresis cycle of the sintered KNL-NTS samples obtained by conventional method at 1100 °C for 2 and 16 h and by microwave technology at 1050 °C for 20 min are shown in Figure 10.

## 4. Discussion

The thermogravimetric analysis of the KNL-NTS milled powder mixture before calcining showed four marked peaks in the derived curve (Figure 2). The first peak appears at 96 °C and corresponds to the loss of water with a weight difference of 8%. The second peak at 192 °C, corresponds with the decomposition reaction of hydrated carbonate to anhydrous carbonate with a 1.7% mass loss. The third and fourth peaks correspond with the decomposition of carbonates to oxides, which occurs in range of the 400–760 °C. From 760 °C no further weight loss occurs, indicating the end of the reaction. The temperature obtained agrees with the calcination temperature reported by other authors, which is in the range of 700–800 °C [22,25]. Therefore, the temperature range between 650 °C and 800 °C was selected for the calcination studies of the powders by conventional and microwave processes.

To study how the heating rate affects the calcination reaction, thermogravimetric analyses (TGA), were performed using different heating rates; 3, 5, 20, 30 and 40 °C·min^−1^ (Figure 3a). The curves agree with a weight loss of 11.7%, very close to the theoretical one. However, it is possible to observe that, as the heating rate increases, a higher temperature is required to complete the reaction. This is confirmed by obtaining the derivative of the curves shown in Figure 3b. The peak at 438 °C with a heating rate of 3 °C·min^−1^ corresponds to the onset of the A_2_CO_3_ to AO_3_ transition and undergoes a shift up to 500 °C when applying 40 °C·min^−1^. The carbonate to oxide transition range, which involves the release of CO_2_ occurs in the range of 400 to 690 °C when heating rates of 3 °C·min^−1^, typical of conventional processing are applied. However, by increasing the heating rate to 40 °C ·min^−1^ the temperature interval increases 30 °C and ends at 800 °C, where the final transformation can be seen [30]. 

Figure 3c shows the differential thermal analysis, DTA, applied at different heating rates. The calcination reaction at 3 °C·min^−1^ presents four endothermic peaks that coincide with the peaks identified by TGA analysis. However, with increasing speed the peaks of the perovskite formation reactions become more endothermic, indicating that the reactions are not completed. New reactions are formed that may be involved with the formation of secondary and or transient phases. At a heating rate of 40 °C·min^−1^, the formation reaction shows exothermic peaks with the formation of different phases. These peaks correspond with those found using the TGA derivative, indicating that the perovskite formation has been destabilized, giving rise to various reactions involving secondary phases. Malic et al. [31] determined that the formation process of KNN-based composites occurs by the diffusion of alkali and oxygen ions into niobium oxide. Furthermore, perovskite formation occurs at the interface between a transient phase and niobium oxide. Thus, different diffusion rates can lead to concentration gradients causing the formation of secondary phases found during sintering.

According to the analysis of the previous figure, X-ray diffraction of the powders calcined by the conventional process at 700 °C for 2 h (heating rate of 30 °C·min^−1^) shows the formation of the perovskite phase with coexistence of orthorhombic and tetragonal symmetries (Figure 4). This is evidenced by the separation between the (002) and (200) peaks located in the range of 2 theta angles 44–47° [32,33]. These peaks are broad due to the crystallite size which is of the order of nanometers and to the presence of the amorphous phase. As the calcination temperature increases (750 and 800 °C), an increase in the crystallinity of the perovskite phase and crystal size is observed [9]. At 800 °C a predominantly orthorhombic structure is shown; this is observed with the pronounced intensity of the peak (002). The presence of secondary phases is not identified. 

The microwave calcined KNL-NTS powders show the presence of peaks corresponding to the formation of the perovskite structure (Figure 5). The dominant geometry is tetragonal, due to a higher intensity of the peak (200) with respect to the peak (002), which is related to the orthorhombic geometry. In addition to the perovskite phase, it is possible to observe the presence of other phases. These can be residues of transitional phases or secondary phases. The identification of these phases can be complex because the main peaks in most of them are located very close to the KNN phases, in addition to the low volume mass fractions, which makes their correct detection difficult. The formation of secondary phases during calcination can be due to various factors. However, in complex systems such as KNL-NTS, the presence of the secondary phases encountered can have a major influence on the heating rate during synthesis on which the kinetics of the piezoelectric compound formation reaction is influenced. Therefore, the application of high heating rates modifies the diffusion capacity of the alkaline elements, promoting the formation of secondary phases. During a conventional synthesis, long processing times produce diffusion processes between the carbonate ions within the structure of the oxides; however, during a short microwave calcination time, the formation of the secondary phases is observed.

The X-ray patterns of KNL-NTS materials sintered at 1100 °C for 2 and 16 h by conventional processing are shown in Figure 6. The peaks found correspond to perovskite formation, without the presence of secondary phases. The separation of peaks (002) and (200) indicate the presence of a cubic structure in the samples processing during 2 h. However, if the dwell time is increased up to 16 h, a coexistence of orthorhombic-tetragonal phases with predominance of the orthorhombic phase is observed. These results are in agreement with the research of Rubio et al. [34], where the evolution of the structure from tetragonal to orthorhombic with sintering time is demonstrated. There are several factors that modify the crystalline structure of KNL-NTS-based composites during sintering: the sintering time at the maximum temperature [34], the concentration gradients of the elements [35], and the addition of dopant elements [22,35], which hinders the reproducibility of the results on the geometry of this system [36].

Figure 7 shows the X-ray diffraction patterns in the 950 to 1050 °C temperature range. It is possible to observe the characteristic peaks of the perovskite phase from 950 °C to 1050 °C. However, at 950 °C the patterns show a distortion along the whole spectrum corresponding to an amorphous phase. This amorphous phase could be due to the relatively low sintering temperature and may correspond to the formation of a solution-precipitation stage leading to the liquid phase [37]. As the sintering temperature increases, the amorphous phase disappears, which could indicate that the coalescence stage has ended. The crystal structure of perovskite is reflected by peaks (002) and (200) and corresponds to a cubic phase [38,39] due to the combination of these peaks.

Microstructure images obtained by FE-SEM of KNL-NTS ceramics sintered by conventional (Figure 8a) and microwave (Figure 8b) processes show cuboidal structures typical of perovskite. Cuboids in the 5–8 µm range with well-defined grain boundaries are visible in the samples obtained by the conventional method with a sintering time of 16 h at 1100 °C. It is also possible to observe residues of the liquid phase typical of sintering of these materials [18,40]. The microstructure of the ceramics processed by microwave at 1050 °C for 10 min shows perovskite cubes with regular geometry, of size close to 2–5 µm; in addition, it is possible to identify the amorphous phase found in the X-ray diffraction patterns. The remains of the liquid phase confirm a sintering mechanism similar to conventional processing. However, there is no heterogeneous heating causing the amorphous phases.

In all cases, the density obtained from the sintered compacts is higher than 90% with respect to the theoretical value determined (Table 1). The densities achieved are very similar, with hardly a 1.4% difference between the highest and lowest values. By the conventional method, the highest density value is obtained when the sintering time is 16 h. Applying this method, densities similar to those reported in the literature by Rubio et al. [19,26,40] (4.47 g·cm^−3^) are obtained. When processing the ceramics by microwave, it is observed that there is no dependence between the sintering time and the density obtained when the ceramics are sintered at 1050 °C. However, it should be noted that microwave sintering only requires a cycle of 40 min to obtain a material with a density similar to that of conventional sintering, which takes approximately 20 h. The energy consumption by microwave is approximately 80% lower than by conventional sintering, which will have an impact on the economic cost of the final material, making it much more competitive at industrial level.

The graphs of dielectric constant as a function of frequency measured for materials sintered by the conventional and microwave processes show dispersions caused by relaxation phenomena due to dipole reorientation (Figure 9). However, the dielectric constant values are higher for samples sintered by the conventional method compared to microwave. This can be attributed to the defects localized by FE-SEM observation.

Figure 10 shows the ferroelectric cycles of the KNL-NTS ceramics sintered by conventional and microwave methods. The KNL-NTS sintered by the conventional method show very similar hysteresis cycles for 2 and 16 h at 1100 °C with saturation polarization (Ps) values at 3500 V of ~17 µC·cm^−2^. The remaining polarization for this voltage is ~15 µC·cm^−2^, while the coercive field is 10 KV·cm^−1^. Dwell time at maximum temperature is not a determining factor in the ferroelectric measurements for this type of sintering.

For microwave sintered samples, the ferroelectric measurements show conduction phenomena, because the saturation polarization is higher than the remanent polarization [36,37,38]. This means that the sample behaves as a conductor and loses its ferroelectric capacity. By increasing the external electric field to 2000 V, the sample becomes a conductor. This same phenomenon occurs in samples sintered at 1050 °C for 30 min.

It has been shown that there are several factors that limit the ferroelectric response of ceramics: low densities [20,39,40], the presence of liquid phase [40] and secondary phases [26]; in addition, the presence of the cubic phase reduces the polarization capacity of the material. Similar phenomena have been obtained by Sridevi et al. [40] in KNN-based materials sintered by multimode microwave. 

## 5. Conclusions

In the present work, as an alternative to the conventional precursor calcination method to obtain piezoelectric materials (K_0.44_Na_0.52_Li_0.04_)(Nb_0.82_Ta_0.10_Sb_0.04_)O_3_, KNL-NTS, microwave-assisted reactive synthesis has been employed. The results suggest that it is possible to synthesize such a compound from a solid-state oxide reaction. However, the microwave heating mechanism, influenced by the high heating rate, alters the obtaining of the pure perovskite phase.

The samples sintered by the conventional method present a piezoelectric constant of 146 pC·N^−1^ and 169 pC·N^−1^ for 2 and 16 h of dwell time. The microwave processed ceramics show low piezoelectric properties ~10 pC·N^−1^ due to low polarization capability. These samples behave as conductors according to the obtained ferroelectric cycles. The use of microwave technology generates secondary phases and defects which restrict the final properties, including ferroelectric properties.

## Figures and Tables

**Table 1:** Physical and electrical properties of KNL-NTS samples.

Sintering Process	Temperature (°C)	Dwell Time (min)	Density (g·cm^−1^)	Relative Density (%)	d33 (pC·N^−1^)
Conventional	1100	120	4.37 ± 0.02	92.6 ± 0.1	145 ± 0.2
Conventional	1100	960	4.41 ± 0.01	93.4 ± 0.1	168 ± 0.2
Microwave	950	10	4.34 ± 0.03	92.0 ± 0.1	11 ± 0.1
Microwave	1000	10	4.35 ± 0.01	92.2 ± 0.1	13 ± 0.1
Microwave	1050	10	4.36 ± 0.02	92.4 ± 0.1	15 ± 0.1
Microwave	1050	20	4.38 ± 0.02	92.8 ± 0.1	10 ± 0.1
Microwave	1050	30	4.38 ± 0.01	92.8 ± 0.1	12 ± 0.1

## Data Availability

Not applicable.
